# Landscape Dynamics in Northwestern Amazonia: An Assessment of Pastures, Fire and Illicit Crops as Drivers of Tropical Deforestation

**DOI:** 10.1371/journal.pone.0054310

**Published:** 2013-01-30

**Authors:** Dolors Armenteras, Nelly Rodríguez, Javier Retana

**Affiliations:** 1 Laboratorio de Ecología del Paisaje y Modelación de Ecosistemas, Departamento de Biología, Facultad de Ciencias, Universidad Nacional de Colombia, Bogotá, Colombia; 2 Centre de Recerca Ecològica i Aplicaciones Forestals i Unitat d'Ecología, Universitat Autònoma de Barcelona, Bellaterra (Barcelona), Spain; University College London, United Kingdom

## Abstract

Many studies have identified drivers of deforestation throughout the tropics and, in most cases, have recognised differences in the level of threat. However, only a few have also looked at the temporal and spatial dynamics by which those drivers act, which is critical for assessing the conservation of biodiversity as well as for landscape planning. In this study, we analyse land cover change between 2000 and 2009 in north-western Colombian Amazonia to identify the interactions between the use of fire, cultivation of illicit crops and establishment of pastures, and their impacts on the loss of forest in the region. Yearly analyses were undertaken at randomly selected sample areas to quantify the average areas of transition of land cover types under different landscape compositions: forest-dominated mosaics, pasture mosaics, fire mosaics, and illicit crop mosaics. Our results indicate that despite the fact that forest areas were well-preserved, deforestation occurred at a low annual rate (0.06%). Conversion to pasture was the main factor responsible for forest loss (the area of pastures tripled within forest mosaics over 8 years), and this process was independent of the landscape matrix in which the forests were located. In fire mosaics, burning is a common tool for forest clearing and conversion to pasture. Thus, forests in fire mosaics were highly disturbed and frequently transformed from primary to secondary forests. The use of fire for illicit cropping was not detected, partly due to the small size of common illicit crops. Forest regeneration from pastures and secondary vegetation was observed in areas with large amounts of natural forest. Overall, assuming the continuation of the observed pasture conversion trend and the use of forest fire, we suggest that our results should be incorporated into a spatially explicit and integrated decision support tool to target and focus land-planning activities and policies.

## Introduction

Rates of forest loss are currently being reported at 0.6% per year at the global level [1], and deforestation remains a prime environmental problem all over the planet. For decades, deforestation in the tropics has been associated with a combination of several economic, demographic, institutional, natural and policy factors, which vary according to the spatial and temporal scale of the area studied [2]–[4]. Forest fires related to slash-and-burn practices, traditionally used for the conversion of forests into agricultural lands and pastures, have been highlighted as a significant factor that degrades and erodes forests and alters their composition and structure [5], [6]. Forest clearance also results in forest fragmentation, increasing the number of forest edges and thus altering sub-canopy humidity conditions, which in turn, increases the susceptibility of the remaining fragments to fire [5]. Grazing area has also increased over recent decades at the expense of forested area, particularly in tropical Latin America [7]. The extensive and expanding production of livestock, spurred by a demand for meat and milk production, which is expected to continue to increase until 2015 [7], [8], is considered another great driver of land use change and deforestation in Latin America. Illicit crops have also recently been noted as a driver of tropical deforestation in tropical biodiversity hotspots [9].

In the case of greater Amazonia, deforestation patterns have been related to the spatial distribution of human-led processes [10]–[13]. The use of fire for land clearing and management is one of the major threats to neotropical forests and one of the most significant sources of carbon emissions to the atmosphere [6], [13], [14]. In the Brazilian Amazon, forest fires represent up to 75% of national CO_2_ emissions [15]. Socioeconomic changes over the last 50 years have led to increasing demands for agricultural land and timber products from tropical forests [16]. This demand has been driven by factors ranging from governments' rural settlement schemes to more enterprise-driven processes such as large-scale agricultural production, for example, the large-scale soybean farming occurring in Brazil [3], [16], [17]. The conversion of forests to livestock pastures has also been identified as an ongoing process in the region [7], similar to the conversion of other natural ecosystems caused by the growing world demand for cereals and oils. The expansion of slash-and-burn agricultural practices is considered one of the primary drivers of changes in tropical land use and land cover [18] and accounts for up to 35% of total deforestation in the Amazon [18].

The north-western Amazon is a tropical region where, in contrast with the Brazilian Amazon, large-scale agricultural practices are still not well established. However, the region hosts a combination of several simultaneous processes, and the synergisms amongst them are responsible for the changes in land use and land cover observed over recent decades. This particular region encompasses extensive and well-preserved tropical forests but also illicit crops, logging activities and pastures [19]. Forest regeneration due to land abandonment has also been identified as an important land cover change occurring in the region [20]. Despite a few recent studies [19], [21] that provide some insights into the location of the colonist frontier in this region, there remains a high level of uncertainty regarding the regional dynamics of land use and land cover change. A further step would be to understand the land use dynamics of the area in order to establish whether and how the human practices that often occur on an annual basis are impacting forest change. The objective of this study is to analyse forest dynamics in north-western Amazonia by studying the interactions between deforestation, the use of fire and the establishment of both illicit crops and pastures for grazing. We will do so by i) looking into land use changes among forests, burned areas, illicit crops and pastures over a nine year period; ii) studying the spatio-temporal patterns of these processes and their relationship with proximity to urban centres; iii) analysing the landscape composition dynamics under four landscape mosaics dominated either by forest, pastures, fire or illicit crops; and finally iv) comparing the general patterns of interactions for the different mosaic types and establishing whether rates of land use change depend on land use pattern. Our hypothesis is that deforestation is primarily driven by pasture conversion mediated by fire and that this transition is well established independent of the type of landscape mosaic and the distance to the colonists' settlements and that, consequently, illicit crops are less important as drivers of deforestation in the region.

## Methods

### Study area

**Figure 1:**
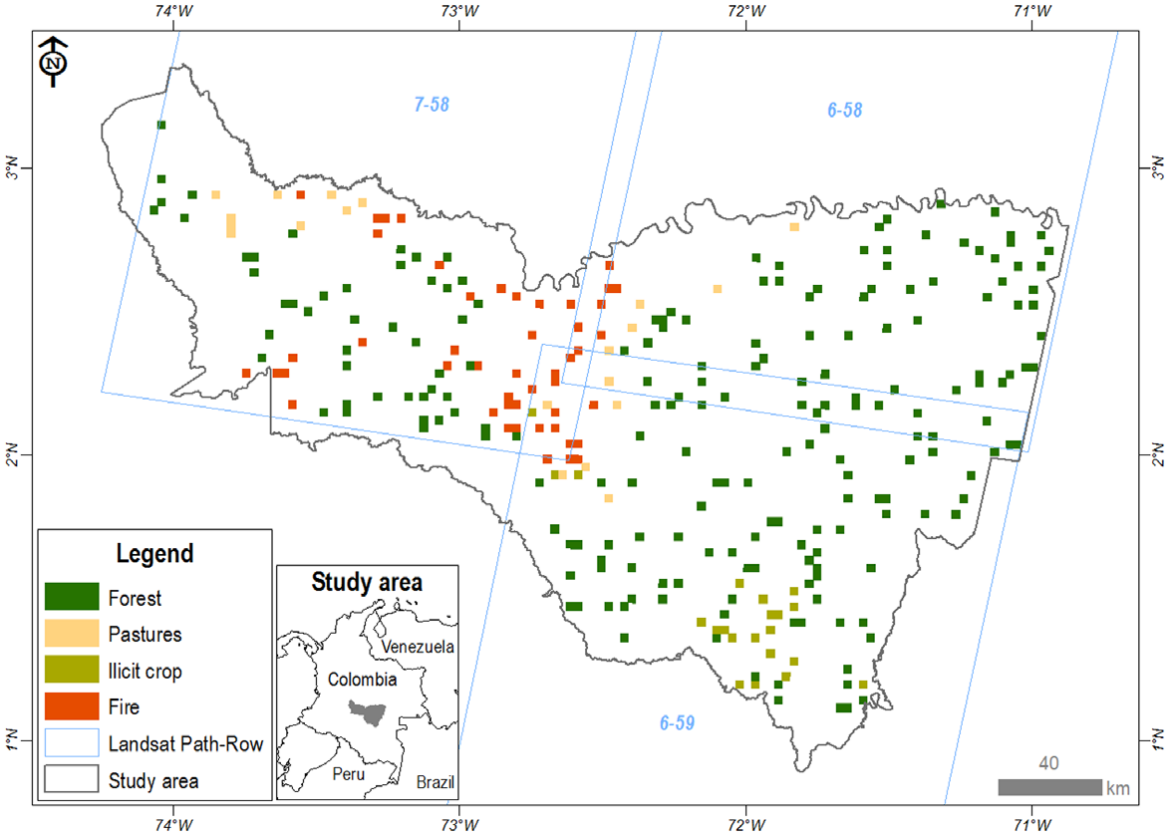
Study Area and distribution of all mosaic types. Landsat Path and Rows indicated.

The study area is located in the north-west region of the Amazon River basin (Figure 1). This region includes two watersheds (Duda and Alto Inirida) within the northern region of the Colombian Amazon, with a total area of 5,413,597 ha. One national park (Sierra de la Macarena), one national natural reserve (Nukak) and 30 indigenous reserves have been established in the area. The region has an average altitude of 100–200 m. The region's climate is tropical, very humid and monomodal, with annual rainfall varying from 2,800 to 3,500 mm and with an average annual temperature of 24.5°C. This region supports high floristic and ecological complexity as a result of its geological, topographic, soil and water gradients. Vegetation types found in the region include several tropical rain forest systems. Due to its location, the region is rich in biodiversity and endemic species.

Economic activities in the region are primarily related to natural resources extractive industries, followed by the establishment of pastures and crops [22]. The forest colonisation process follows the course of navigable rivers and roads. Contrary to what occurs in the Brazilian Amazon, human occupation in Colombian Amazonia does not follow a fishbone pattern since road construction that often is the origin of this type of pattern is not yet one of the main deforestation drivers in the study area [11]. Cultivation of illicit crops (mainly coca, *Erythroxylum coca*) has been the main economic and transformation driver of the region in recent decades, and 17% of the coca recorded in Colombia in 2009 was located in the study area [23]. Livestock is primarily concentrated near municipalities with ongoing development of infrastructure and roads.

### Dataset

#### 1 Land use and land cover

The study area was analysed at two levels. We used the entire study area, which we called the regional level, to provide a regional land use and land cover change context. Additionally, to have comparable units, avoid spatial autocorrelation and facilitate the statistical analyses, we divided the area of the two watersheds into 3×3 km windows (6000 windows). We eliminated the windows without 100% land cover (180) and then randomly selected 291 windows (Figure 1), a sample representing 5% of the total area. We refer to those windows as the local level.

This research was conducted using a series of Landsat 5 TM and Landsat 7 ETM (Path/Rows 6/58, 6/59 and 7/58) collected mostly in the months of December, January, February and March from the years 2000 to 2009 (Table S1). Images were acquired at no cost from the United States Geological Survey (USGS) archive (http://glovis.usgs.gov/). Two years, 2001 and 2009, were chosen for the regional level classification due to information constraints (i.e., the availability of cloudless images for the whole region). At the local level, a classification was performed for each year from 2000 to 2009 except for two years for which cloudless images were not available (2002 and 2007). All images were rectified to Magna-Sirgas (the Coordinate Reference System officially used in Colombia) with an error of less than 1 pixel. We classified land cover into the following categories: i) forests; ii) pastures, iii) burned areas, including both recently burned and post-fire areas; iv) secondary forests, including regenerating vegetation; v) other (clouds, urban areas, roads, etc.); and vi) coca cultivation.

As a first step, we classified the burnt area separately for each scene for each year. All scenes were classified individually, with a mask for bodies of water, rocky outcrops and urban areas to minimise errors, given the similar spectral responses of these cover types to burnt areas. We then used the normalised burn ratio (NBR) developed by Key and Benson [24]. This index was calculated using the formula NBR = (TM 4−TM 7)/(TM 4+TM 7) where TM 4 and TM 7 refer to Landsat TM bands 4 and 7, respectively. The NBR threshold values used for the analysis were based on the mean and standard deviation, with pixels considered burned when their index value fell within the range of 2 standard deviations. The accuracy of the identification of burned areas was verified using CBERS images from the period 2004–2008 (http://www.dgi.inpe.br/CDSR/) being the accuracy over 82% in all cases, the lowest in 2004 .

The second step was to acquire annual information on coca cultivation based on existing spatial data from the yearly global illicit crop monitoring program of the United Nations Office on Drugs and Crime [25], represented in Colombia by the Integrated System for Illicit Crops Monitoring project, or SIMCI (Sistema Integral de Monitoreo de Cultivos Ilicitos, [23]). This information comes from a mixed manual and supervised classification combined with intensive field work, with a reported accuracy of over 89% for all years [23].

As a third step, we classified land cover for all scenes using ENVI Software (Version 4.5), applying a mixed technique including both an unsupervised algorithm (ISODATA) and visual interpretation, using bands 3, 4, and 5 and the NDVI (Normalised Difference Vegetation Index). Fifteen initial spectral classes were created and were grouped into 4 categories: forest, pasture, secondary vegetation (the separation of this class is based on spectral response, and it includes secondary forests, secondary vegetation, disturbed forests primarily caused by abandoned pasture and disturbed forests caused by logging or similar activities) and others (clouds, urban areas, roads and bodies of water). Accuracy assessments were performed only for 2001 and 2006 and followed the methodology described by Meidinger [26]. They were conducted by visually checking the map against aerial photographs, high resolution images (SPOT, CBERS) and other detailed maps available for the area, such as the national CORINE Land Cover map set [27]. The final accuracies for the 2001 and 2006 maps were 93% and 89%, respectively.

A final annual classification dataset for each scene was created by adding the burnt area and the coca cultivation classes to the unsupervised classification. To weaken the visually observed salt-and-pepper effect, a majority filter using a 3×3 pixel moving window was applied as a smoothing technique to remove speckles. Visual examinations before and after smoothing indicated no observable loss of data.

#### 2 Active fires

Aside from the burned area algorithm applied in the land cover classification, we also utilised the fire hotpots series MODIS (Moderate Resolution Imaging Spectroradiometer) product processed in the Collection 5.1 temporal thermal analysis active fire dataset [28]. This dataset was not incorporated into the land cover classification but was instead used to analyse the spatial pattern of fires over time for the entire region. The daily dataset spanning December 2000 to December 2009 was downloaded from FIRMS (Fire Information for Resource Management System: Archiving and Distributing MODIS Active Fire Data, Collection 5; [28]). The dataset detected active fires based on the thermal signature of fire using a contextual algorithm [29].

#### 3 Analysis

We utilised ArcGIS (ESRI) to conduct all digital spatial analyses. As mentioned, we analysed the data at two scales, the first being a regional scale for both watersheds, covering a total area of 4,538,109 ha (84% of the two watersheds). For this regional extent, we first quantified annual changes for all six land use types between 2001 and 2009. Change rates (*r*, %) were calculated using the formula suggested by Puyravaud [30]:
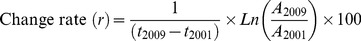
(1)where A_2001_ and A_2009_ are the forest (or other land use) areas in hectares in the years t_2001_ and t_2009_, respectively.

Transitions between land use categories were also evaluated at the regional extent, using transition matrices (indicating the proportion of land use by category in 2000 (rows) that changed to a land use category in 2009 (columns). We also analysed, for the entire region, the change over time in the areas of coca cultivation and fire hotspots (2000–2009), calculating the number of occurrences and their average distance to San Jose del Guaviare (an urban centre and one of the settlements established as a reference point for the colonisation frontier). In the case of coca, we also computed total cultivation area and the average size of the crops.

**Figure 2:**
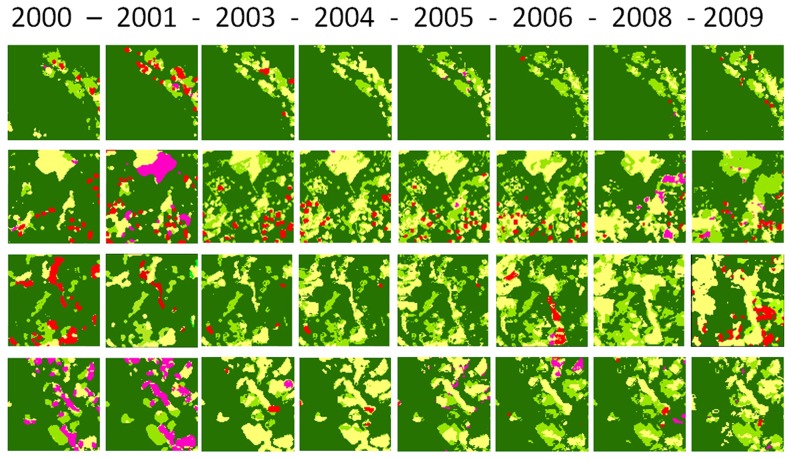
Land use changes during the period of analysis in four of the 291 windows (3 km×3 km), one of each mosaic type. A, Forest mosaics; B, Pasture mosaics; C, Fire mosaics; and D, Illicit crop mosaics. Forest (dark green), secondary vegetation (light green); pastures (yellow), burned area (red); illicit crops (purple).

At the local level, a simple random sample of 3×3 km windows (291) represented 5.7% of the total study area. For each window, we quantified the annual areas of land use/cover categories (forest, coca, pastures, burned area, and secondary vegetation) using 2000 as the base year. For the purpose of the analysis, we then categorised the selected windows that were post-stratified into four groups according to their matrices: (a) Forest mosaics, those windows with >90% forested area (202 windows); (b) Burned area mosaics, with >5% burned area (48 windows); (c) Pasture mosaics, with >10% pasture (20 windows); and (d) Coca mosaics, with >5% coca crops (21 windows). These four classes were mutually exclusive; we prioritised burned areas and coca when assigning classes. No windows were found to belong to more than one category. We analysed the composition of the landscape (i.e., the proportion of the landscape occupied by each land use type and its variation over time) for all 291 windows, and also compared the average compositions of the four mosaic types. Figure 2 illustrates an example of each type of mosaic for the 291 windows.

The annual transition rates between land uses amongst the four types of mosaics (forests, pastures, illicit crops and fire) were compared using ANOVA tests. We carried out a different ANOVA analysis for each transition between pairs of land uses, that is, 25 analyses (5×5 transitions between land uses). As there were many comparisons, we thought to use the Bonferroni correction, but it was not necessary because most of the analysis results were not significant (see below). The values of the different transitions between years were the replicates (*N* = 7).

## Results

### 1 Land use changes in the region

The study area was dominated by forests that in 2001 represented 83.7% of the land area as well as, to a lesser extent, pastures (8.0% of land area; Table 1). Loss of forest cover was low for the whole study region over this time period (19,600 ha), and thus, the annual rate of deforestation was also low (0.06%). Pasture coverage increased considerably from 2001 (8.0%) to 2009 (10.3%), with an annual rate of change of 2.77%, while burned areas expanded with an annual rate of change of 2.60%. There was also a significant reduction in the area of illicit crops (−11.4%; Table 1).

**Table 1:** Land uses in 2001 and 2009 (ha and %) and change rate for the entire study area.

Land use	Area (ha) in 2001	% in 2001	Area (ha) in 2009	% in 2009	Annual Change Rate (r)
Forest	3,799.684	83.73	3,780.083	83.30	−0.06
Burned Area	52,340	1.15	66,130	1.46	2.60
Secondary vegetation	218,000	4.80	211,993	4.67	−0.31
Pastures	364,790	8.04	467,866	10.31	2.77
Illicit crops	27,973	0.62	10,019	0.22	−11.41
Others	75,324	1.66	2,019	0.04	-

The probabilities of the transition matrix between 2001 and 2009 (Table 2) showed that a high proportion of forests (94.8%) and pastures (75.5%) maintained the same land cover (what is termed persistence, expressing the permanence of each cover type over time) over the 8 yr time period analysed. Few pixels changed on an annual basis, and thus year-to-year persistence was high in all categories, with the lowest rate of persistence being illicit crops at 87.7%. After 8 years, a large proportion of both burned areas and illicit crops had converted to pastures (53.3% and 50.4%, respectively).

**Table 2:** Land use transition probability matrix (P) for the entire study area over an 8-year time step.

	Forest	Burned Area	Secondary vegetation	Pastures	Illicit crops
Forest	**0.948**	0.007	0.020	0.023	0.002
Burned Area	0.227	**0.114**	0.115	0.533	0.004
Secondary vegetation	0.329	0.050	**0.374**	0.242	0.004
Pastures	0.095	0.058	0.087	**0.755**	0.003
Illicit crops	0.319	0.050	0.114	0.504	**0.013**

Rows and columns indicate land uses in 2001 and 2009, respectively.

**Figure 3:**
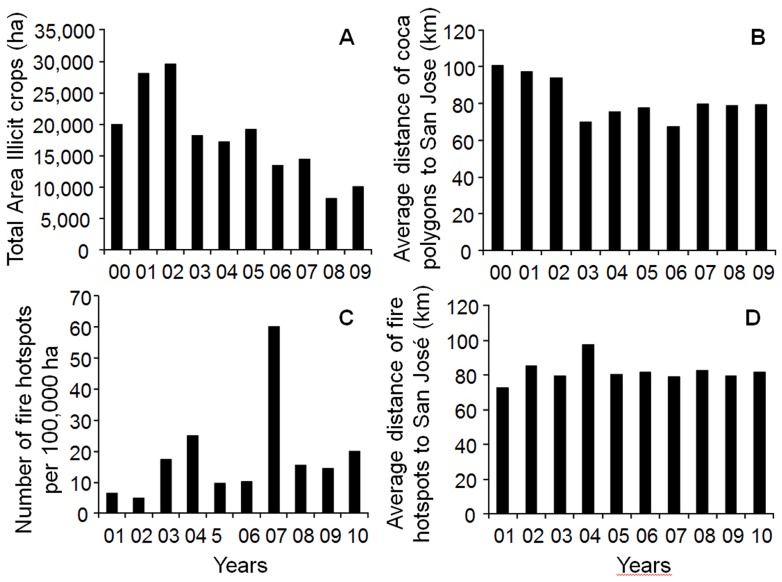
Variations between 2000 and 2009 at the regional level. In: A, Total coca area in ha; B, Average distance of coca fields from San Jose del Guaviare (km); C, Number of fire hotspots detected per 100,000 ha; D, Average distance of fire hotspots from San Jose del Guaviare (km).

The year-by-year analysis of the spatio-temporal pattern of illicit crops confirmed the above-mentioned steady reduction in the total area of illicit crops for the region (Figure 3A). The average distance of coca crops from San Jose de Guaviare did not show the expected pattern of increasing distance over time following the expansion of the colonist frontier, but the distance tended to decrease over time, indicating that, on average, illicit crops were being cultivated closer to the urban centre (Figure 3B). The number of fire hotspots in the study area varied over the years (Figure 3C), and 2004 (25.2 fire hotspots/100,000 ha) and especially 2007 (60.2 fire hotspots/100,000 ha) were the years with highest numbers of hotspots. The average distance of fire hotspots to San Jose did not show any discernible pattern (Figure 3D).

### 2 Changes in landscape composition over time in the four landscape mosaics

**Figure 4:**
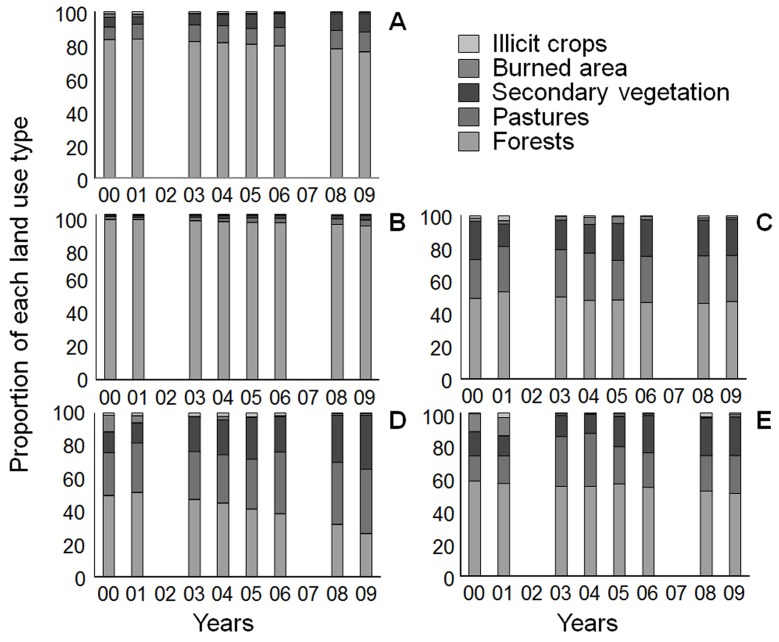
Proportion of each land use type over time in windows of all mosaics. (A); Forest mosaics (B); Pasture mosaics (C);Fire mosaics (D); and Illicit crop mosaics (E).

Based on our results (Figure 4A), we observed that when all windows (291) were considered, the trend over time followed the same general pattern as that for the entire study region. Thus, forests remained the dominant land use over time with a small reduction in area (from 83.4% in 2000 to 76% in 2009), and both pastures and secondary vegetation increased their proportion of the landscape (from 7.6% and 5.6% in 2000 to 11.9% and 11.1% in 2009, respectively). Illicit crops and burned areas decreased from 1.34% and 1.97% of the sampled windows in 2000 to 0.55% and 0.35% in 2009, respectively.

The comparison of the four mosaics revealed a different pattern in each case (Figure 4). In forest-dominated mosaics (Figure 4B), the pattern was similar to the general pattern (Figure 4A), with a higher proportion of forests dominating the landscape but also with a slight reduction from 97.2% in 2000 to 93% in 2009. The area of pastures tripled in 9 years (from 0.75% to 2.65% in 2009) and secondary vegetation doubled (from 1.5% to 3.5% in 2009). Contrary to the general trend of reduction of illicit crops, illicit crops increased in forest-dominated landscapes from 0.27% in 2000 to 0.43% in 2009. In pasture mosaics (Figure 4C), forests represented 49.1% of land area in 2000 and 47% in 2009, while pastures increased from 24.1% in 2000 to 28.6% in 2009 and secondary vegetation decreased from 23.1% in 2000 to 21.7% in 2009. Both illicit crops and burned areas decreased in this type of mosaic. In fire-dominated mosaics (Figure 4D), the pattern was similar to the pasture mosaic, with a reduction in forest cover, an increase in pastures and an increase of secondary vegetation. Both burned area and illicit crops decreased in this mosaic type. Finally, in the illicit crop-dominated mosaics (Figure 4E), forest cover decreased from 58.2% in 2000 to 50.7% in 2009 and burned areas from 11% to 1.4% in 2009, while secondary vegetation and pastures both increased in area from 2000 to 2009.

### 3 Rates of land use change in the four landscape mosaics

**Figure 5:**
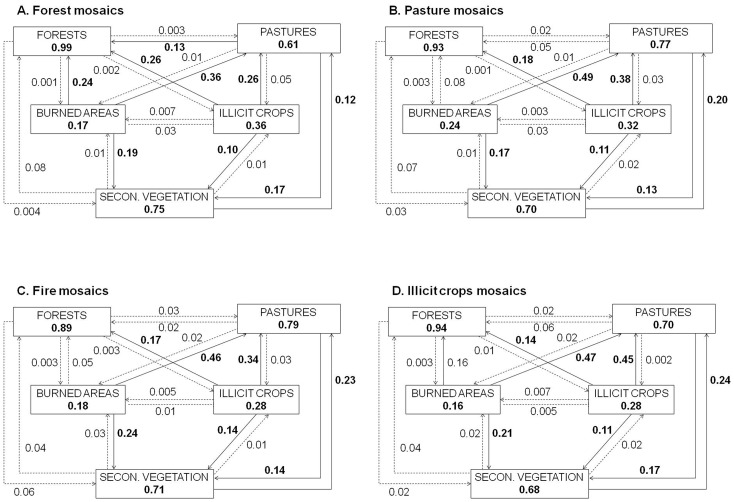
Average land use changes of the yearly values in Forest mosaics. (A); Pasture mosaics (B); Fire mosaics (C); and Illicit crop mosaics (D). Numbers in bold indicate transitions >10%.

The transition matrix of land use changes for the four types of mosaics in the 2000–2009 period (illustrated in Figure 5) indicated a high persistence of forest cover in all cases, but especially in the forest mosaics (99%, Figure 5a). Pastures also had high persistence in both fire (79%, Figure 5c) and pasture mosaics (77%, Figure 5b), with lower persistence in forest-dominated mosaics (61%, Figure 5a). Persistence of secondary vegetation was similar in all mosaics. Recurrence of burned areas was high in the pasture mosaics (24%, Figure 5b) and lower in the illicit crops mosaics (16%, Figure 5d). The persistence of illicit crops was low (28–36%) across all mosaics. Conversion to pasture from other land use types was high in all mosaic types. Conversion of previously burned areas ranged between 36% in forest-dominated mosaics (Figure 5a) to 49% in pasture mosaics (Figure 5b). Transition from illicit crops to pastures was also significant, occurring at rates from 26% in forest mosaics (Figure 5a) to 46% in illicit crop (Figure 5d) mosaics. Transitions from secondary vegetation to pastures, although less common than the already–mentioned transitions, ranged from a rate of 12% in forest mosaics (Figure 5a) to 24% in illicit crop mosaics (Figure 5d).

Concerning the differences in transitions between land uses in the four mosaics types, only two transition types showed significant differences (*P*<0.05), whereas two more were marginally significant (*P*<0.10). The first significant transition, from forests to secondary vegetation (F = 4.3, *P* = 0.014), was considerably higher in the fire mosaics (7%) than in the pasture mosaics (3.7%), illicit crop mosaics (3%), and especially the forest mosaics (0.4%). The opposite pattern was observed in the transition from pastures to forests (F = 3.4, *P* = 0.032), with significantly higher values in the forest mosaics (13.5%) than in the illicit crop (6.8%), pasture (5.9%) and fire mosaics (4.1%). The transition from illicit crops to forest was marginally significant (F = 2.4, *P* = 0.088), with higher values for the forest mosaics (26.4%) than for the fire (18.7%), pasture (17.7%) and especially the illicit crop mosaics (14.3%). The transition from illicit crops to secondary vegetation was also marginally significant (F = 2.4, *P* = 0.088), with higher values for the fire mosaics (15.1%) than for the other three types of mosaics (values between 10.0 and 11.4%).

## Discussion

### 1 Land use changes in the region

The low annual deforestation rate reported for the study area is similar to other deforestation rates reported for colonist frontier areas in the region associated with shifting subsistence agriculture, few settlements and low population density (0.04%/year [19]). The presence of one national protected area (Macarena) and one National Nature Reserve (Nukak) in the region is likely to act as an effective buffer against deforestation, as previously reported [20]. However, our calculated deforestation rate for the study region is far from what Wassenaar et al. [7] projected. Those authors previously predicted a higher percentage of forest conversion to pastures for Latin America, with a projected 8.1% loss in forest cover for Colombia in 2010 and conversion to pastures representing 68% of the total deforested area for the country. The relatively low loss of forest but still-significant increase in pastures we found between 2001 and 2009 suggests that deforestation in the region is mainly the result of the clearing of forests for pastures, but is also a complex process involving several land uses. This region has also been strongly influenced by the cultivation of illicit crops for a long time [19], [25], and the reduction in extent of this crop might, on first consideration, appear to be a positive development because illicit crop production has also been identified as a factor driving deforestation [9].

The results obtained from the land use change matrix (Table 2) indicate that not only the changes in area of land use types but also their persistence and dynamics must be considered. Altogether, the results highlight the strong land use dynamics between illicit crops, fire and pastures over the 8 year period considered. The high percentage of burned areas that became pastures after 8 years confirms the use of fire as a management technique to prepare soil for cattle grazing. Illicit crops were reduced in extent, but after 8 years, basically no illicit crop polygons remained in their initial locations (1.3%), and most of the original illicit crops were converted to pastures. The reduction in total area and the low persistence of these illegal activities indicate that there is a spatial dynamic of the cultivation of illicit crops moving to new areas. In contrast, pastures, once they are established, remain stable (75.5%). This suggests that the area might be undergoing a transformation from a pattern of colonisation to a transition occupation model characterised by the production of large livestock. In this model, the expansion of the colonist frontier is less influenced by the cultivation of coca crops, and some human settlements become permanent [19]. The lack of change over time in the average distance of forest fires from San Jose gives some indication that vegetation burning activities do not occur progressively farther from human settlements. As such, it lends support to the idea that large-scale cattle production is becoming established in the area, leading to the permanent and recurring use of fire for pasture preparation. There is also a clear influence of climatic events, such as the dry years in 2004 and 2007, on the number of fires detected, as stated in previous studies [31]. No clear pattern of increasing distance of illicit crops to San Jose del Guaviare was found, which could be a reflection that proximity to forest borders or even an edge effect could be more important than distance to a human settlement such as the one considered in this study. A recent report mentioned that 30% of illicit crop parcels are found within 10 km of the forest border and that up to 80% are found within 30 km [25].

### 2 Landscape composition dynamics and rates of land use change in different landscape mosaics

The regional analysis provides an overview of land use changes between 2000 and 2009, but does not reflect the year-by-year dynamics in the region. In the second level of analysis (of the 3×3 km mosaics on an annual basis), the persistence of forests and the low levels of deforestation follow the same general pattern described above in the analysis of the whole study area. However, several differences arise, in particular the fact that illicit crops increased in forest mosaics. This pattern begins to explain where illicit crop farmers are moving to because one would have expected forest loss to be higher in those mosaics that were already highly transformed into pastures in 2001. After the introduction of pastures in previously forested areas, further exploitation transforms secondary vegetation into pastures, indicating that cattle grazing is becoming the predominant economic activity for those areas. Forests are becoming scarce in the fire mosaics, most likely due to the intense and recurrent use of fire, which might increase forest susceptibility to fire and lead to rapid forest loss. All pasture, fire and illicit crop mosaics are steadily becoming more pasture-dominant. This pattern is less strong in illicit crop mosaics, most likely due to the abandonment of illicit crops and their displacement to new areas, but the proportion of forest loss is much higher in illicit crop mosaics than in pasture and fire mosaics.

Comparative results between average land use transitions for the four mosaic types may be useful for understanding the dynamics of deforestation and the establishment of pastures in different types of matrix-dominated landscapes. On the one hand, forests in the region are still relatively intact, but are slowly being eroded due to a pasture-led deforestation process. That process is stronger in fire-dominated landscapes, where forest persistence is low and forest is starting to become a scarce land use type. In this context, we detected the degradation of primary forest to secondary forest, which is most likely an indication of recurring fire activity in the process of conversion to pastures, as reported for other parts of the Amazon [13]. Without any doubt, one of the strongest land use changes is from burned areas to pastures, confirming the use of fire to prepare soils, no matter where the conversion occurs. Additionally, the transition from illicit crops to pastures reaffirms the idea that land abandonment and displacement is most likely due to illicit crop eradication efforts, which force people to relocate these types of activities elsewhere, thus promoting further deforestation and fragmentation [9]. Land occupation in the region is based on the clearing of primary forest and on pastures, and the expansion and intensification of agriculture does not yet exist. This might be partly due to the illicit crop economy, which is a temporary exploitation of a crop strongly linked to armed and illegal groups [9]. The observation of forest regeneration in forest mosaics reaffirms the idea that the abandonment of agricultural lands in remote areas where forests are not strongly fragmented can lead to forest regeneration. Finally, land use history (including vegetation recovery) in the region is highly dynamic, and thus should be taken into account when producing estimates of carbon emissions from tropical deforestation and forest fires.

### 3 Conclusions

Our research provides a regional insight into the land use dynamics of one of the most biodiverse regions of north-western Amazonia, where livestock production, fires and illicit crops are causes of deforestation at different levels. Pasture conversion is the primary land use change occurring and is by far the main cause of deforestation, while human-related fires for the purpose of land clearing and for soil preparation are also common in the area. A recent study in Bolivia found similar patterns of greater conversion of forest to pasture than to coca [32]. In contrast, illicit crops have strong temporal and spatial dynamics and are a driver of forest fragmentation through the perforation of forests with small and transient crop areas of less than 0.6 ha. Over time, the cultivation of illicit crops advances into intact forests, promoting the further destruction of tropical forests in the region. However, some of these areas are later abandoned, leading to forest regeneration. Indeed Amazonian land use systems have fluctuated over the long term with agriculturalist fire reduction and post contact reforestation, for instance occurring after the European conquest [33]. The fluctuations we have observed might probably have some role in global CO_2_ decline as has happened in the past [34]. However the ecological and environmental consequences of this process are largely unknown.

Land use knowledge such as provided in this paper may serve to target and focus conservation and development policies. These studies are effective for highlighting new colonisation frontier areas, where forest cover is at risk of slowly disappearing. The data gathered in this research also highlight the contribution of fire usage to deforestation as well as pasture preparation. Future estimates of carbon emissions resulting from Colombian deforestation would be improved by including an understanding of land use dynamics in estimates of carbon emissions resulting from fire, and by differentiating between deforestation fires and pasture maintenance fires. Post-clearing land use also needs to be taken into account in estimates of carbon losses, as has been suggested for the Brazilian Amazon [13]. The largest difference between this region and the rest of the Amazon is that large-scale agriculture has not yet been established. However, the study area might be at the beginning of an unplanned trend towards more intensive land management, as is occurring in other areas of the Amazon [13].

## Supporting Information

Table S1 **Landsat images used in this study.**
(DOCX)

## References

[1] Hansen MC, Stehman SV, Potapov PV (2010) Quantification of global gross forest cover loss. PNAS 107 19: 8650–8655. 20421467 10.1073/pnas.0912668107 PMC2889354

[2] Geist HJ, Lambin EF (2002) Proximate causes and underlying driving forces of tropical deforestation. BioScience 52: 143–150.

[3] Rudel TK (2006) Shrinking tropical forests, human agents of change and conservation policy. Conserv Biol 20: 1604–1609. 17181795 10.1111/j.1523-1739.2006.00532.x

[4] Kindermann G, Obersteiner M, Sohngen B, Sathaye J, Andrasko K, et al (2008) Global cost estimates of reducing carbon emissions through avoided deforestation. PNAS 105: 10302–10307. 18650377 10.1073/pnas.0710616105 PMC2483236

[5] Cochrane MA (2003) Fire science for rainforests. Nature 421: 913–919. 12606992 10.1038/nature01437

[6] Cochrane MA, Laurance WF (2008) Synergisms among fire, land use, and climate change in the Amazon. Ambio 37 7–8: 522–527. 19205173 10.1579/0044-7447-37.7.522

[7] Wassenaar T, Gerber P, Verburg PH, Rosales M, Ibrahim M, et al (2007) Projecting land use changes in the Neotropics: The geography of pasture expansion into forest. Global Environ Change 17: 86–104.

[8] United Nations Environment Programme – UNEP (2010) Latin America and the Caribbean: environment outlook. GEO LAC 3. Mexico: United Nations Environment. 114 p.

[9] Dávalos LM, Bejarano AC, Hall MA, Correa HL, Corthals AP, et al (2011) Forests and drugs: coca-driven deforestation in global biodiversity hotspots. Environ Sci Technol 45: 1219–1227. 21222455 10.1021/es102373d

[10] Soares-Filho BS, Assuncao RN, Pantuzzo AE (2001) Modelling the spatial transition probabilities of landscape dynamics in an Amazonian colonization frontier. Bioscience 51: 1059–1067.

[11] Armenteras D, Rudas G, Rodríguez N, Sua S, Romero M (2006) Patterns and causes of deforestation in the Colombian Amazon. Ecol Indicators 6: 353–368.

[12] Arce-Nazario J (2007) Human landscapes have complex trajectories: reconstructing Peruvian Amazon landscape history from 1948 to 2005. Landscape Ecol 22: 89–101.

[13] Morton DC, Defries RS, Randerson JT, Giglio L, Schroeder W, et al (2008) Agricultural intensification increases deforestation fire activity in Amazonia. Global Change Biol 14: 2262–2275.

[14] Aragao LEOC, Malhi Y, Barbier N, Lima A, Shimabukuro Y, et al (2008) Interactions between rainfall, deforestation and fires during recent years in the Brazilian Amazonia. Philos Trans R Soc Lond B Biol Sci 363: 1779–1785. 18267907 10.1098/rstb.2007.0026 PMC2373892

[15] Fearnside PM (2007) Uso da terra na Amazônia e as mudanças climáticas globais. Braz J Ecol 10: 83–100.

[16] Rudel TK, Defries R, Asner GP, Laurance WF (2009) Changing drivers of deforestation and new opportunities for conservation. Conserv Biol 23 6: 1396–1405. 20078640 10.1111/j.1523-1739.2009.01332.x

[17] Vera-Diaz MDC, Kaufmann RK, Nepstad DC, Schlesinger P (2008) An interdisciplinary model of soybean yield in the Amazon Basin: The climatic, edaphic, and economic determinants. Ecological Economics 65 2: 420–431.

[18] Metzger JP (2002) Landscape dynamics and equilibrium in areas of slash-and-burn agriculture with short and long fallow period (Bragantina region, NE Brazilian Amazon). Landscape Ecol 17: 419–431.

[19] Rodriguez N, Armenteras D, Molowny-Horas R, Retana J (2012) Patterns and trends of forest loss in the Colombian Guyana. Biotropica 44 1: 123–132.

[20] Armenteras D, Rodríguez N, Retana J (2009) Are conservation strategies effective in avoiding the deforestation of the Colombian Guyana Shield? Biol Conserv 142: 1411–1419.

[21] Etter A, McAlpine C, Wilson K, Phinn S, Possingham H (2006) Regional patterns of agricultural land use and deforestation in Colombia. Agric Ecosyst Environ 114: 369–386.

[22] Ariza E, Ramírez MC, Vega L (1998) Atlas cultural de la Amazonia colombiana: La construcción del territorio en el siglo XX. Bogotá: Instituto Colombiano de Antropología. 219 p.

[23] United Nations Office on Drugs and Crime - UNODC (2009) Colombia: Monitoreo de Cultivos de Coca 2009. Bogotá: UNODC. 112 p.

[24] Key CH, Benson NC (2006) Landscape assessment: ground measure of severity, the Composite Burn Index; and remote sensing of severity, the Normalized Burn Ratio. In DCLutes; REKeane; JFCaratti; CHKey; NCBenson; et al.. (2006). FIREMON: Fire Effects Monitoring and Inventory System. USDA Forest Service, Rocky Mountain Research Station, Ogden, UT Gen Tech Rep RMRS-GTR-164-CD: LA 1-51.

[25] United Nations Office on Drugs and Crime (UNODC) (2010) Transformación socioeconómica y biofísica asociadas con cultivos ilícitos en la región Sur del Meta'Guaviare (1999–2009). Bogotá: SIMCI-UNODC. 153 p.

[26] Meidinger DV (2003) Protocol for accuracy assessment of ecosystem maps. Tech. Rep. 011. Victoria, Canada: Ministry of Forests, Forest Science Program. 23 p.

[27] Instituto Geografico Agustin Codazzi, Comision Corografica (2007) Ecosistemas continentales, costeros y marinos de Colombia. Bogotá: Imprenta Nacional de Colombia. 275 p.

[28] Davies DK, Ilavajhala S, Wong MM, Justice CO (2009) Fire information for resource management system: archiving and distributing MODIS active fire data. IEEE Trans Geosci Remote Sens 47 1: 72–79.

[29] Giglio L, Descloitres J, Justice CO, Kaufman YJ (2003) An enhanced contextual fire detection algorithm for MODIS. Remote Sens Environ 87: 273–282.

[30] Puyravaud JP (2003) Standardizing the calculation of the annual rate of deforestation. For. Ecol. Manage 177: 593–596.

[31] Armenteras-Pascual D, Retana-Alumbreros J, Molowny-Horas R, Roman-Cuesta RM, Gonzalez-Alonso F, et al (2011) Characterising fire spatial pattern interactions with climate and vegetation in Colombia. Agricultural and Forest Meteorology 151 3: 279–289.

[32] Bradley AV, Millington AC (2008) Coca and colonists: quantifying and explaining forest clearance under coca and anti-narcotics policy regimes. Ecology and Society 13 1: 31.

[33] Nevle RJ, Bird DK (2008) Effects of syn-pandemic fire reduction and reforestation in the tropcial Americas on amospheric CO2 durign the European conquest. Palaeogeography, Palaeoclimatology, Palaeoecology 264: 25–38.

[34] Kaplan JO, Krumhardt KM, Ellis EC, Ruddiman WF, Lemmen C, et al (2010) Holocene carbon emissions as a result of anthropogenic land cover change. The Holocene 21 5: 775–791.

