# Supplementary material for: Landscape Dynamics in Northwestern Amazonia: An Assessment of Pastures, Fire and Illicit Crops as Drivers of Tropical Deforestation
Source: PLoS One. 2013 Jan 30;8(1):e54310. doi: 10.1371/journal.pone.0054310 (PMC3559686; doi:10.1371/journal.pone.0054310)
Supplement: Table S1 — Landsat images used in this study. (DOCX) [file pone.0054310.s001.docx]

**Table S 1.**

| **Path/Row** | | **Acquisition date** |
| --- | --- | --- |
| 6/58 | | 01/05/2000 |
|  | | 01/03/2001 |
|  | | 01/13/2003 |
|  | | 02/01/2004 |
|  | | 01/26/2005 |
|  | | 02/14/2006 |
|  | | 04/16/2008 |
|  | | 12/28/2009 |
| 6/59 | 01/05/2000 | |
|  | 04/09/2001 | |
|  | 12/28/2002- 01/13/2003 - | |
|  | 01/16/2004 | |
|  | 01/26/2005 | |
|  | 02/06/2006 - 02/14/2006 | |
|  | 01/11/2008 - 02/04/2008 | |
|  | 02/14/2009 - 12/28/2009 | |
| 7/58 | 01/05/2000 | |
|  | 03/12/2001 | |
|  | 01/04/2003 | |
|  | 01/23/2004 - 02/24/2004 | |
|  | 12/24/2004 | |
|  | 01/12/2006 - 02/13/2006 | |
|  | 01/02/2008 | |
|  | 02/14/2009 - 02/21/2009 | |
